# Mode of Coronary Revascularization and Short term Clinical Outcomes in Patients with Chronic Kidney Disease

**DOI:** 10.12669/pjms.306.5540

**Published:** 2014

**Authors:** Ashique Ali Khoso, Khawar Abbas Kazmi, Saqiba Tahir, Hasanat Sharif, Safia Awan

**Affiliations:** 1 Ashique Ali Khoso, Senior Instructor, Section of Cardiology, Department of Medicine, Aga Khan University, Karachi, Pakistan.; 2 Khawar Abbas Kazmi, Professor and Section Head of Cardiology, Department of Medicine, Aga Khan University, Karachi, Pakistan.; 3 Saqiba Tahir, Medical Student, Aga Khan University, Karachi, Pakistan.; 4 Hasanat Sharif, Associate Professor and Section Head of Cardiothoracic Surgery, Aga Khan University, Karachi, Pakistan.Department of Surgery, Aga Khan University Hospital,; 5 Safia Awan, Statistician, Department of Medicine, Aga Khan University, Karachi, Pakistan.

**Keywords:** Chronic kidney disease, Creatinine clearance, Coronary revascularization, Percutaneous coronary intervention, Coronary artery bypass graft

## Abstract

***Background and Objective:*** Percutaneous coronary intervention (PCI) and coronary artery bypass graft (CABG) surgery are two alternative methods for coronary revascularization, but it remains controversial as which one is associated with lower risks of worse clinical outcomes for chronic kidney disease (CKD) patients. We determined the mode of coronary revascularization (PCI vs. CABG) which is associated with lower risk of mortality and morbidity in CKD patients.

***Methods:*** In this cross sectional study, 159 patients with CKD were enrolled from single center of coronary revascularization at Aga Khan University Hospital Karachi between January 2012 and August 2013. All patients with CKD underwent PCI or CABG. The primary outcome was in-hospital composite of death, myocardial infarction (MI), or stroke. We evaluated which mode of coronary revascularization was associated with reduced risks of clinical outcomes.

***Results:*** Out of 159 patients with CKD, 85 (53.5%) received PCI and 74 (46.5%) received CABG. The primary finding of this study is that more patients with moderate to severe CKD underwent PCI and more patients with mild to moderate CKD underwent CABG. In both these categories, no difference was observed in clinical outcomes. There are few factors like age, ST- elevation myocardial infarction (STEMI), non-ST elevation myocardial infarction (NSTEMI) and number of coronary artery disease predicted PCI as treatment strategy in patients with moderate to severe CKD.

***Conclusion:*** Patients with moderate to severe CKD have similar rates of short term clinical outcomes whether they underwent PCI or CABG. Therefore, PCI can be acceptable and less invasive treatment option alternative to CABG, particularly in patients with moderate to severe CKD.

## INTRODUCTION

Chronic Kidney Disease (CKD) is a major public health issue with the prevalence continuing to escalate, in part due to increasing diabetes and hypertension.^1^ In patients undergoing coronary revascularization for either stable coronary artery disease or acute myocardial infarction, CKD is one of the strongest risk factors for short- and long-term mortality.^2^ Mortality, myocardial infarction (MI), repeat revascularization, and bleeding complication rates are all increased after coronary revascularization compared with that of patients with normal renal function.^3^ CKD is an independent predictor of incident stroke, myocardial infarction (MI), and all-cause mortality.^4^

There is evidence that coronary revascularization reduces the cardiac mortality and improves prognosis compared to medical treatment for CKD patients with CAD.^5^ With regard to evidence of best mode of coronary revascularization, percutaneous coronary intervention (PCI) and coronary artery bypass grafting (CABG) are two alternative methods, but it remains controversial as which one is associated with reduced major adverse cardiac and cerebral events (MACCE), reduced risk of worsening kidney function and need of hemodialysis and reduced in-hospital stay for CKD patients.

Previous studies suggested that CKD is associated with increased mortality after CABG^6^ perhaps because such patients have longer postoperative mechanical ventilation time, higher postoperative bleeding rates and transfusion requirements, and increased length of hospital stay. PCI in patients with CKD is also high-risk due to their increased incidence of worsening kidney function, restenosis, and mortality.^7^ The increased risk occurs even with mild renal insufficiency; with a doubling of mortality at one year.^8^ To the best of our knowledge, there is paucity of prospective study results on clinical outcomes of CKD in patients undergoing coronary revascularization as well as optimal strategy for coronary revascularization which is associated with lower risks of cardiovascular mortality and morbidity in resource constraint countries of South-Asian region particularly Pakistan.

The aim of this study was to determine the mode of coronary revascularization (PCI vs. CABG) in CKD patients, which is associated with less major adverse cardiac cerebral events (MACCE) and lower risk of worsening kidney function and need of hemodialysis and minimum in-hospital stay.

## METHODS

In this cross sectional study, 159 patients with CKD were enrolled from single center of coronary revascularization at Aga Khan University Hospital Karachi between January 2012 and August 2013. The mode of revascularization included Percutaneous Coronary Intervention (PCI) [Patients were treated with one or more stents but excluding POBA (Plain Old Balloon Angioplasty)] and Coronary Bypass Grafting (CABG). Patients were excluded if there was severe anemia or liver disease. In total, 159 patients with CKD formed the study sample (122 men, 37 women and mean age 65±9.6 [range 38-88] years). Patients were stratified into three groups based on Creatinine Clearance (CrCl). Baseline demographics, clinical and angiographic features were recorded for each patient. This study was reviewed and approved by the institutional ethical review committee of Aga Khan University Hospital. All patients had serum creatinine measured 24-48 hours before the revascularization. Renal function was assessed by the creatinine clearance (CrCl) estimated using the Cockcroft-Gault formula.^9^ Patients with estimated creatinine clearance (CrCl) ≤ 90 ml/min were defined as having Chronic Kidney Disease (CKD), consistent with CKD stage 2 of the National Kidney Foundation classification^10^ and these patients were classified into 3 CrCl groups: <30ml/min (n= 59), 30-59ml/min (n= 79) and 60-89ml/min (n=21). All patients who underwent coronary revascularization (PCI or CABG) were observed during the hospital stay after the procedure. During the hospital stay, major adverse clinical outcomes (MACCE) including all-cause of death, new onset myocardial infarction, and stroke were recorded. Within the first 7 days after revascularization, MI was defined by appearance of new abnormal Q-wave (according to Minnesota Code^11^ plus a ratio of serum creatinine kinase MB (CK-MB) iso- enzyme to total cardiac enzyme >0.1 or a Creatinine kinase MB elevated at least 3-fold the upper limit of the normal range. After 7 days of the revascularization, abnormal Q-waves or enzymatic changes were enough to diagnose MI.^12^ Stable angina was defined according to the system of the Canadian Cardiovascular Society (CCS).^13^ Unstable angina was defined according to the Braunwald classification.^14^ The number of diseased coronary vessels was defined as number of coronary arteries with luminal diameter narrowing ≥70% and the left main coronary artery stenosis ≥50% was considered to be 3-vessel coronary artery disease (3V-CAD). Left anterior descending (LAD) proximal lesion was defined as stenosis ≥70% in the proximal half of the LAD. Long lesion was defined as stenosis > 20mm. Ostial lesion was defined as stenosis within 3mm of the origin of the vessel. Complete revascularization was defined if there was no remaining stenosis ≥70% in the coronary artery luminal diameter ≥ 2mm.

A descriptive analysis was performed for demographic and clinical characteristics and results are presented as mean ± standard deviation for quantitative variables and numbers (percentages) for qualitative variables. Participants with CKD treated with CABG and PCI with stenting were assessed by using the Chi-square test or Fisher exact test where appropriate. For contrasts of continuous variables, Independent t-test was used to assess the difference of means. In univariate analysis, compared the association of PCI with clinical outcomes and other covariates. Logistic regression analysis was performed to assess factors that predict favorable outcome. Patients were stratified into three groups based on Creatinine Clearance (CrCl). Comparisons among groups were made by pearson chi-square test for categorical variables and ANOVA test for variance for continuous variables. All analyses were conducted by using the Statistical package for social science SPSS (Release 19.0, standard version, copyright © SPSS; 1989-02). All p-values were two sided and considered as statistically significant if < 0.05.

## RESULTS

In this study of 159 patients with CKD, (mean age 65±9.6 (38-88) years, 122 (76.7%) of them were male and the indication for revascularization were non-ST-elevation MI (69, 43.4%), ST-elevation MI (34, 21.4%), stable angina (31, 19.5%) and unstable angina (27, 17%). The mode of coronary revascularization was PCI (85, 53.5%) or CABG (74, 46.5%).

 Baseline characteristics like hypertension, diabetes mellitus, current smoking history, history of ischemic or hemorrhagic stroke, valvular heart disease, prior MI, prior revascularization, left ventricular ejection fraction (LVEF<40%) and indications for revascularization were not significantly different among 3 groups of CKD. All three groups of CKD had multivessel coronary artery disease. Almost one third of patients in severe CKD group (33.9%) were on long term hemodialysis before the revascularization, whereas only few number of patients, 2 (9.5%) and 3 (3.8%) in the mild and moderate CKD groups respectively were on long term hemodialysis before revascularization. 

The evidence of left main lesion, LAD proximal lesion, ostial lesion, chronic total occlusion, in-stent restenosis, long lesion or complex lesion was almost similar among the 3 groups of CKD. In the severe CKD group, more patients underwent PCI whereas, in the mild to moderate groups of CKD, more patients underwent CABG. Though the rate of failed PCI was similar among the 3 groups of CKD but complete revascularization was more evident in mild to moderate CKD. The baseline creatinine (p<0.001) and creatinine on admission (p<0.001) were higher in the PCI group compared to CABG group (2.7±1.7), (3.4±2.3) and (1.7±0.9), (1.8±1.04) respectively. More importantly, the choice of mode of revascularization among moderate to severe CKD group (CrCl 30-59 ml/min and CrCl <30ml/min) was PCI as compared to CABG which was mode of revascularization in mild to moderate CKD group (CrCl 30-59 ml/min and CrCl 60-89ml/min). More patients with CKD already on hemodialysis were in the PCI group as compared to CABG group 21 (24.7%) and 4 (5.4%) with p value of 0.001.

In terms of indications of revascularization, CABG was treatment of choice in patients with stable angina whereas PCI was the choice of mode of revascularization in STEMI and NSTEMI. Serum creatinine after 48 hours of procedure was higher in PCI group as compared to CABG group (3.2 ± 2.1 and 2.5 ± 0.9) respectively. Along with this, 12 (14.1%) patients in the moderate to severe CKD group underwent hemodialysis and CRRT (continuous renal replacement therapy) after the procedure and all of them were in the PCI group; 6 of them were already on hemodialysis prior to procedure. In-hospital stay was 5.4 ± 3.9 and 10.6 ± 6.9 days with p˂0.001 for PCI and CABG group respectively (Table-I). There was no difference in the frequency of the individual short term outcomes of stroke, MI, or death between treatment groups PCI vs. CABG (Table-II). There are few factors like age, STEMI, NSTEMI, prior revascularization, complete revascularization and number of coronary artery disease which predicted PCI as treatment strategy in patients with moderate to severe CKD (Table-III).

## DISCUSSION

The primary finding of this observational study is that more patients with moderate to severe CKD underwent PCI and more patients with mild to moderate CKD underwent CABG. In both of these categories, clinical outcomes were similar. In the recent meta-analysis done by Chen YY and his colleagues, high heterogeneity in short term mortality was found among the studies included in the meat analysis. Chen YY states in his study that worse kidney function in the beginning was associated with worse post-procedure outcomes, but PCI still had reduced short term mortality as compared to CABG in dialysis dependent patients.^15^ Interestingly, this finding in our study is opposite to study done by Zhang Q et al. who had shown that patients with lowest CrCl underwent CABG while patients with normal creatinine or mild renal insufficiency underwent PCI.^16^

**Table-I:** Characteristics of patients with CKD after PCI and CABG

	**Total** **(n=159)**	**PCI** **(n=85)**	**CABG** **(n=74)**	**p value**
Age (years)		67±9	63±9	0.003
Male	122(76.7%)	63 (74.1%)	59(79.7%)	0.45
Female	37 (23.3%)	22(25.9%)	15(20.3%)	
Hemoglobin	11.5 ± 1.76	11.4±1.8	11.6±1.6	0.66
WBC	11.1 ± 5.1	12.4±6.2	9.5±2.8	˂0.001
Baseline creainine(mg/dl)	2.3 ± 1.5	2.7±1.7	1.7±0.9	˂0.001
Creatinine on admission(mg/dl)	2.6 ± 2.0	3.4±2.3	1.8±1.04	˂0.001
Creatinine Clearance (ml/min)				
˂30	59(37.1%)	51(60%)	8(10.8%)	<0.001
30-59	79(49.7%)	28(32.9%)	51(68.9%)	
60-89	21(13.2%)	6(7.1%)	15(20.3%)	
Current smoking history	159(100%)	5(5.9%)	13(17.6%	0.02
Hypertension	144(90.6%)	77(90.6%)	67(90.5%)	0.99
Diabetes mellitus	108(67.9%)	60(70.6%)	48(64.9%)	0.99
History of ischemic stroke	16(10.1%)	11(12.9%)	5(6.8%)	0.27
History of hemorrhagic stroke	1(0.6%)	0(0.0%)	1(1.4%)	0.36
Valvular heart disease	34(21.4%)	19(22.4%)	15(20.3%)	0.84
Prior MI	59(37.1%)	36(42.4%)	23(31.1%)	0.18
Prior kidney disease	157(98.7%)	85(100%)	72(97.3%)	0.21
with dialysis	25(15.7%)	21(24.7%)	4(5.4%)	0.001
without dialysis	134(84.3%)	64(75.3%)	70(94.6%)	0.001
Peripheral vascular disease	1(1.2%)	1(1.2%)	0(.0%)	0.99
Prior revascularization	40(25.2%)	31(36.5%)	9(12.2%)	<0.001
LVEF <40%	77(48.4%)	41(48.2%)	36(49.3%)	0.99
Indications for revascularization				
Stable angina	31(19.5%)	5(5.9%)	26(35.1%)	<0.001
Unstable angina	27(17%)	10(11.8%)	17(23%)	0.08
NSTEMI	69(43.4%)	44(51.8%)	25(33.8%)	0.02
STEMI	34(21.4%)	26(30.6%)	8(10.8%)	0.003
Number of diseased vessels				
1	25(15.7%)	23(27.1%)	2(2.7%)	<0.001
2	45 (28.3%)	28(32.9%)	17(23.3%)	<0.001
3	88(55.3%)	34(40%)	54(74%)	˂0.001
Creatinine after 48 hours of procedure	2.9±1.7	3.2 ± 2.1	2.5 ± 0.9	0.004
Required Heamodialysis after procedure	159(100%)	12 (14.1%)	0 (0%)	0.001
Length of stay(days)	7.8 ± 6.8	5.4 ± 3.9	10.6 ± 6.9	˂0.001

MI, myocardial infarction; PCI, percutaneous coronary intervention; CABG, coronary artery bypass graft;

**Table-II:** Clinical outcomes among patients with CKD after PCI or CABG

	**PCI**	**CABG**	**OR (95% CI)**	**p value**
MACCE	18 (21.2%)	14 (18.9%)	1.15[0.52-2.51]	0.72
Death	10 (11.8%)	8(10.8%)	1.10[0.41-2.95]	0.85
Cardiogenic	2(2.4%)	3(4.1%)	1.31[0.21-8.10]	0.76
Non cardiogenic	8(9.4%)	5(6.8%)	1.43[0.44-4.59]	0.54
MI	9 (10.6%)	8 (10.8%)	0.97[0.35-2.67]	0.96
Stroke	1(1.2%)	2(2.7%)	2.33[0.20-26.26]	0.49

OR, odds ratio; CI, confidence interval; MACCE, major adverse cardiac cerebral events.

Other abbreviations see Table-I.

**Table-III:** Multivariate Analysis of Factors predicting PCI among CKD patients

	**OR (95% CI)**	**p value**
Age	1.06 (1.001-1.14)	0.04
NSTEMI	18 (3.22-100)	0.001
STEMI	8.54 (1.46-50)	0.01
Prior revascularization	21 (3.59-119.2)	0.001
Complete revascularization	0.004 (0-0.04)	<0.001
Number of disease vessels		
2	0.03(0.005-0.25)	0.001
3	0.005 (0.001-0.04)	<0.001

OR, odds ratio; CI, confidence interval; Other abbreviations see Table-I.

Similar findings were present in the study done by Ix JH et al in which he had shown that participants with mild to moderate CKD undergoing coronary revascularization had similar rates of MI, stroke, or death whether they underwent PCI with multivessel stenting or CABG.^17^ The observational studies done in the past showed conflicting results about the implications of CKD on clinical outcomes after coronary revascularization. Szczech et al. found the adjusted estimated 2-year survival to be 51.9% after PCI and 77.4% after CABG in patients with renal insufficiency.^18^ In the study with greater utilization of stents in PCI patients, Reddan et al. found a survival advantage with CABG compared with PCI which appeared to increase as renal function declined.^19^ In contrast to these studies, PCI provided survival benefit in comparison to medical management in patients with mild to moderate renal insufficiency.^19^ Nevertheless, Rubenstein et al found more promising short- and long-term outcomes using advances in interventional cardiology such as stents and debulking devices.^20^ In retrospective study of 1,654 patients with a glomerular filtration rate of <60ml/min revealed that PCI yielded better results than medical therapy and CABG in renal insufficiency patients with acute coronary syndromes.^21^

Another important point in our study is that patients with mild and moderate CKD received CABG while patients with moderate to severe CKD received PCI, this might indicate the selection bias on part of operator. It means patients with severe kidney disease and acute coronary syndromes are undergoing PCI. Our study has also shown that patients who were undergoing PCI were older and they had higher baseline and admission serum creatinine. Therefore there were few factors like age, STEMI, NSTEMI, and number of coronary artery disease which predicted PCI as treatment strategy in patients with moderate to severe CKD. But in our study short term clinical outcomes are not different among two treatment groups. Because worse kidney function in the beginning was associated with worse post-procedure outcomes. These equivalent rates of stroke, new-onset MI, or cardiogenic and non-cardiogenic death between two treatment groups among patients with CKD indicate that PCI can be acceptable and less invasive treatment option alternative to CABG, particularly in patients with moderate to severe CKD. Despite the new advancements in interventional cardiology, including drug eluting stents(DES) and bioabsoable stents, distal protection devices, and use of newer antithrombotic agents and marked progress in CABG in terms of on-pump CABG, optimal treatment strategy in patients with CKD undergoing coronary revascularization is still debatable. The primary limitation of this study is that it was not randomized study and it had shown only short term clinical outcomes. It also had relied on estimated creatinine clearance calculated through the Cockcroft-Gault formula, which is an imprecise measure of kidney function.

## CONCLUSION

The present study is perhaps the first of its kind in this part of South-Asian region to prospectively evaluate mode of coronary revascularization and the clinical outcomes in patients with CKD. Our results have demonstrated that patients with moderate to severe CKD have similar rates of short term clinical outcomes whether they underwent PCI or CABG. Our results also confirm that CKD is a significant risk factor for patients undergoing coronary revascularization. PCI can be acceptable and less invasive treatment option alternative to CABG. Further randomized controlled trials with longer follow up are required to establish the optimal strategy for coronary revascularization in patients with CKD in this part of the world.

## Authors Contribution:

***Khawar Abbas Kazmi:*** Conceptualization of Project and Final Approval of the manuscript.

***Ashique Ali Khoso and Saqiba Tahir: ***Data Collection and Literature Search.

***Ashique Ali Khoso and Safia Awan: ***Statistical Analysis.

***Ashique Ali Khoso, Khawar Abbas Kazmi and Hasanat Sharif: ***Drafting, Revision and Writing of the Manuscript.
